# A Social Media Study on the Associations of Flavored Electronic Cigarettes With Health Symptoms: Observational Study

**DOI:** 10.2196/17496

**Published:** 2020-06-22

**Authors:** Long Chen, Xinyi Lu, Jianbo Yuan, Joyce Luo, Jiebo Luo, Zidian Xie, Dongmei Li

**Affiliations:** 1 University of Rochester Rochester, NY United States; 2 Princeton University Princeton, NJ United States; 3 University of Rochester Medical Center Rochester, NY United States

**Keywords:** e-cigarette, social media, eHealth

## Abstract

**Background:**

In recent years, flavored electronic cigarettes (e-cigarettes) have become popular among teenagers and young adults. Discussions about e-cigarettes and e-cigarette use (vaping) experiences are prevalent online, making social media an ideal resource for understanding the health risks associated with e-cigarette flavors from the users’ perspective.

**Objective:**

This study aimed to investigate the potential associations between electronic cigarette liquid (e-liquid) flavors and the reporting of health symptoms using social media data.

**Methods:**

A dataset consisting of 2.8 million e-cigarette–related posts was collected using keyword filtering from Reddit, a social media platform, from January 2013 to April 2019. Temporal analysis for nine major health symptom categories was used to understand the trend of public concerns related to e-cigarettes. Sentiment analysis was conducted to obtain the proportions of positive and negative sentiment scores for all reported health symptom categories. Topic modeling was applied to reveal the topics related to e-cigarettes and health symptoms. Furthermore, generalized estimating equation (GEE) models were used to quantitatively measure potential associations between e-liquid flavors and the reporting of health symptoms.

**Results:**

Temporal analysis showed that the Respiratory category was consistently the most discussed health symptom category among all categories related to e-cigarettes on Reddit, followed by the Throat category. Sentiment analysis showed higher proportions of positive sentiment scores for all reported health symptom categories, except for the Cancer category. Topic modeling conducted on all health-related posts showed that 17 of the top 100 topics were flavor related. GEE models showed different associations between the reporting of health symptoms and e-liquid flavor categories, for example, lower association of the Beverage flavors with Respiratory compared with other flavors and higher association of the Fruit flavors with Cardiovascular than other flavors.

**Conclusions:**

This study identified different potential associations between e-liquid flavors and the reporting of health symptoms using social media data. The results of this study provide valuable information for further investigation of the health effects associated with different e-liquid flavors.

## Introduction

### Background

Electronic cigarette (e-cigarette) is an electronic nicotine delivery system (ENDS) that vaporizes electronic cigarette liquid (e-liquid), a solution that contains a number of substances [1]. E-cigarette is promoted as a tobacco substitute, which usually delivers nicotine, flavorings, and other additives via an inhaled aerosol. Since the first device publicly marketed in 2003, the e-cigarette market has experienced a significant growth in prevalence among all age groups [2].

The flavored e-cigarette market has grown extremely rapidly and has become increasingly popular among teenagers and young adults. A recent study identified 7764 distinct e-liquid flavors in 2014, and the number doubled to 15,586 in 2017 [3]. Adolescents and young adults prefer electronic cigarettes (e-cigarettes) over conventional tobacco because they are more affordable, accessible, convenient, and, especially, come with a variety of flavors [1]. A recent examination of the Population Assessment of Tobacco and Health (PATH) Wave 2 data found that more than 75% of youth e-cigarette users used a flavored e-cigarette with Fruit being the most popular e-cigarette flavor category, followed by the Candy/Sweet flavor category [4]. Nearly two-thirds of the adult e-cigarette users vape (ie, the behavior of inhaling vaporized aerosol) a flavored e-cigarette with Menthol or Mint as the most popular flavor category, followed by the Fruit flavor category [4]. The 2019 National Youth Tobacco Survey showed that current e-cigarette consumption among high school students increased from 1.5% in 2011 to 27.5% in 2019, with an estimated 72% of high school e-cigarette users using a flavored e-cigarette [5]. In addition, the number of e-cigarette users in middle school (grades 6-8) increased from 3.3% in 2017 to 10.5% in 2019, with nearly 60% of the middle school e-cigarette users using a flavored e-cigarette [5].

With the increasing popularity of e-cigarettes, it becomes important to understand the health symptoms associated with e-cigarettes and flavored e-cigarette consumption. There were claims that e-cigarettes are 95% safer than conventional tobacco, and nicotine released into the environment, if the e-cigarette liquid contains any, is negligible [6]. A previous study has shown that nonsmokers, such as children and pregnant women, had cardiovascular and other diseases due to passive vaping [7]. E-cigarette flavorings could damage human blood vessel cells in a laboratory environment, even in the absence of nicotine [8]. A number of studies have shown the association of vaping with some respiratory diseases or symptoms, including wheezing and asthma [9-12]. The cinnamon-flavored vaping products had the most potent cytotoxicity, leading to significantly decreased cell viability, increased reactive oxygen species levels, and other health symptoms [8]. Exposure to ENDS aerosol resulted in decreased metabolic activity and cell viability, whereas flavors significantly affected the toxicity of ENDS aerosol, with menthol, coffee, and strawberry flavors having the most significant impact on the overall cytotoxicity of e-cigarette products [13].

### Objectives

E-cigarettes and e-cigarette use experiences are widely discussed on social media. Thus, the association of user-reported health risks with e-cigarette flavorings could be explored from social media posts. A recent review on e-liquid flavors using the JuiceDB data from 2013 to 2017 found that Fruity and Sweet were the two most popular e-liquid flavors and have more positive sentiments from users [14]. However, possible associations of e-liquid flavors with health risks have not been well investigated using social media data. Therefore, we aimed to examine probable associations between e-liquid flavors and the reporting of health symptoms using social media data through a data-driven approach.

Reddit was chosen as our main online data source. Reddit is a platform for any user (older than 13 years) to discuss, connect, and share their experiences and opinions online [15]. Besides not allowing the unwelcome content (eg, illegal, sexual, and confidential information), Reddit prohibit users from asking for votes or engaging in vote manipulation. As one of the biggest social media platforms in the United States, Reddit operates like a forum with divided communities called *subreddit*s, such that each one focuses on a specific topic such as sports and politics. Empirically, Reddit contains fewer advertisements and promotions but more self-reporting discussions from vapers (ie, people who vape), making it a desired source to reflect users’ experiences and opinions [16]. In addition, discussions about e-liquid flavors have been shown to be one of the most prevalent topics about e-cigarettes on Reddit [17], with Fruit, Sweet, and Cream being the most prevalent flavors discussed among Reddit users [18]. A keyword list was generated by combining the keywords used in a previous e-cigarette–related social media study [19] and was used to filter out non-e-cigarette posts. To analyze the textual contents and users’ opinions, topic modeling (Latent Dirichlet Analysis [LDA]) and sentiment analysis were conducted. We applied generalized estimating equation (GEE) models to quantify the potential associations between e-liquid flavors and major related health symptoms based on the e-cigarette–related discussions from Reddit users. Our study provides valuable information about the potential health effects of different e-liquid flavors, which could guide future research on flavored e-cigarettes.

## Methods

### Data Collection

We obtained the Reddit posts between January 01, 2013, and April 30, 2019, from pushshift.io [20]. We curated a list of e-cigarette–related terminologies (eg, vape, e-cig, e-liquid, and e-juice) from a prior study [19] on related topics as a preliminary list for keyword matching to generate an e-cigarette–related dataset. To account for typos and abbreviations, we applied Frequent Itemset Mining (FIM) based on the first iteration of the keyword-filtered dataset. FIM accepts all posts as input, and it outputs the combinations of most frequently appearing words in the dataset. We then manually selected high-frequency keywords to enrich the initial keyword list.

To investigate the e-cigarette–related health concerns discussed by Reddit users, we further generated a health-related subset (referred to as the health subset) from the e-cigarette dataset. A preliminary health keyword list was curated from a related prior study [21]. The list mostly comprised medical terminologies; thus, we applied FIM after filtering the preliminary list to include relevant conversational terms that frequently appear in our dataset. Similarly, a flavor-related subset (referred to as the flavor subset) was generated by using a flavor-related keyword list. The flavor keywords were generated by crawling the top e-cigarette manufacturers’ websites to collect brand names, e-cigarette products’ marketing names, and key ingredients.

All datasets were validated and denoised. We manually examined the noisy keywords (eg, blue, hard, and life can be flavor names or have other semantic meanings) from the keyword lists and removed the related posts if such keywords result in low precision during the filtering process. For keywords that were less semantically generic and noisy, we applied an additional filter to clean the data. For example, the flavor keyword “tobacco” contributes to a noisy flavor subset, which contains posts only about conventional tobacco products. We discovered a filter rule that filters out most of the undesired data while maintaining a decent level of recall to ensure the completeness of the dataset, by selecting 10 flavor-related keywords such as “flavor” and “e-liquid,” which were found to frequently appear when users talk about tobacco-flavored e-cigarettes rather than conventional tobacco products. As a result, posts were classified as tobacco flavor-related when at least one sentence of the post contains both tobacco and one of the 10 flavor-related keywords. To examine the accuracy of our filter, we selected our top 10 flavor-related keywords, randomly picking 100 posts for each keyword. We then highlighted the sentences that contained the flavor-related keyword and manually examined whether the sentences were about discussions on e-cigarette–related topics. The accuracy of filtering was then calculated and is shown in Multimedia Appendix 1. Most flavor-related keywords have an accuracy between 0.8 and 0.9, which can be considered as clean.

Finally, we curated an e-cigarette keyword list of 20 terms (“e-cig,” “e-cigs,” “ecig,” “ecigs,” “electroniccigarette,” “ecigarette,” “ecigarettes,” “vape,” “vapers,” “vaping,” “vapes,” “e-liquid,” “ejuice,” “eliquid,” “e-juice,” “vapercon,” “vapeon,” “vapefam,” “vapenation,” and “juul”) and constructed the e-cigarette dataset consisting of 2,865,467 posts from 623,258 unique users. For data preprocessing, all URLs, email addresses, and non-English posts were removed to form a cleaner dataset. We created the health subset by applying 144 health keywords from nine health categories (Multimedia Appendix 1) and obtained 337,482 health-related posts from 138,448 unique users after filtering. Similarly, the flavor subset was generated with a flavor list of 1229 flavors (Multimedia Appendix 1) from 123 e-cigarette brands under 7 flavor categories including Fruit, Menthol or Mint, Tobacco, Sweet, Beverage, Mixed, and Other, containing 446,440 posts from 111,869 unique users.

### Temporal Analysis

A temporal analysis was conducted to investigate longitudinal changes in the discussions about e-cigarettes on Reddit from January 2013 to April 2019. In addition, discussions about e-liquid flavors and health symptoms associated with e-cigarettes were examined using temporal analysis. A temporal trend for the percentage of Reddit posts for each reported health symptom was calculated as the number of Reddit posts mentioning each reported health symptom divided by the total number of e-cigarette related Reddit posts in each month.

### Sentiment Analysis of Posts With Health Symptom Category Mentions

Sentiment analysis is a contextual analysis of sentences and paragraphs, which can extract subjective attitudes and opinions from the source documents. The Valence Aware Dictionary and sEntiment Reasoner (VADER) was used as the sentiment analyzer to extract users’ opinions when discussing about health symptoms in each category on Reddit [22]. We calculated the sentiment score for each Reddit post and the average sentiment score for each health category. As some health-related keywords have negative sentiments (eg, headache), such keywords were replaced by a sentiment-neutral word “X.” Such a process better reflects users’ opinions about e-cigarettes. Sentiment propensity was then computed for each post with the suggested threshold from VADER. We classified posts with sentiment scores between +0.05 to +1.00 as positive posts, posts with sentiment scores between −0.05 to +0.05 (not including −0.05 and +0.05) as neutral, and posts with sentiment scores between −1.00 to −0.05 as negative. We normalized the numbers of positive, neutral, and negative posts by the total number of posts in each category to reflect the distribution of sentiment results. In addition, we compared the proportions of positive and negative posts within each health category using two proportion Z-tests in statistical analysis software R to determine the significant differences between the proportion of positive sentiments and the proportion of negative sentiments for each health category. We set the significance level for all tests at 5% and adjusted the original *P* values using the Bonferroni method to control for multiple testing error rate.

### Topic Modeling

Topic modeling, specifically the LDA model, was applied to identify e-cigarette–related topics that were most frequently discussed [23]. LDA is a generative model for unsupervised topic modeling that can be regarded as a three-layer Bayesian distribution of words (or terms), topics, and documents such that each word in a document is expected to be allocated to a specific topic, where words in each topic are given a certain weight representing the possibility of appearance.

We applied topic modeling to the health subset. After data cleaning, all uppercase characters were converted to lowercase to ensure consistency, and words were lemmatized using spaCy to its stem form because different tenses will not be considered in the model. In addition, we removed stop-words (eg, you, I, and) using the Natural Language ToolKit.

As the LDA model considers each word’s possibility of occurrence in a topic, we converted frequent phrases into one word so that a phrase can be considered as an *element* of a document, rather than a combination of separated words. For example, “throat” and “hit” are often mentioned together; thus, topics that contains both words as a “term” could be regarded as one element. We processed the dataset to find frequent bigrams (eg, panic attack, blood pressure, and lipid pneumonia) and trigrams (eg, food drug administration) using the Gensim package and then converted them into a single term.

### Associations Between E-Liquid Flavors and Health Symptoms

To determine possible associations between e-liquid flavors and the reporting of health symptoms, we applied GEE models based on binomial distributions with logistic link functions. The within-user correlations were considered through the compound symmetry variance-covariance matrices under the GEE model framework. The GEE models were conducted using the statistical analysis software SAS v9.4 (SAS Institute Inc).

From the e-cigarette dataset, we selected posts with only one flavor-related keyword and at least one health-related keyword. If one selected post contained more than one health keyword, each health-flavor keyword pair was regarded as one unique entry. In total, 3336 entries were included in the dataset. From the GEE models, the probability of comentioning each flavor category with each health category was estimated, and the Tukey method [24] was used to adjust for multiplicity in pairwise comparisons between flavors.

The estimated probabilities of associations represent the probabilities that one flavor category and one health symptom category were comentioned by the same user. By performing pairwise comparisons, significant differences in the probabilities of association were identified within each health category. A heatmap was created to highlight the probabilities of associations between e-cigarette flavors and the reporting of health symptoms. All tests were two-sided with a significance level of 5%.

## Results

### Classification of Health Symptoms

To better understand the potential association of e-liquid flavors with the reporting of health symptoms, we grouped 144 health keywords collected from Reddit into nine health categories, such as Respiratory, Neurological, and Cardiovascular (Multimedia Appendix 1). By counting the number of times mentioned in 337,482 Reddit posts, we found that Respiratory was the most mentioned health category (94,691/337,482, 28.06%), followed by Other (75,102/337,482, 22.25%), Mouth (44,212/337,482, 13.10%), Neurological (33,650/337,482, 9.97%), Throat (33,151/337,482, 9.82%), Psychological (31,041/337,482, 9.20%), Cardiovascular (15,460/337,482, 4.58%), and Digestive (6876/337,482, 2.04%) categories. Cancer was the least mentioned health category on our Reddit dataset at only 0.97% (3299/337,482).

### Temporal Analysis of Health Symptom Category Mentioned on Reddit

**Figure 1:**
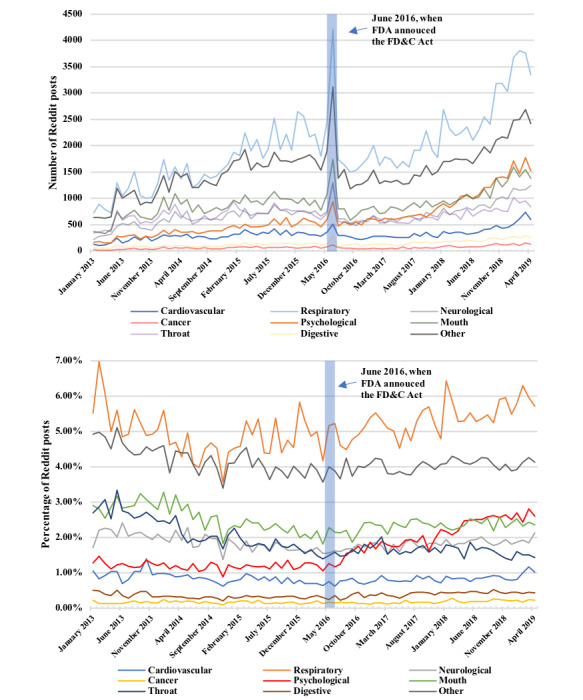
Monthly health-related posts count and proportion of health-related posts (normalized to electronic cigarette–related posts) from January 2013 to April 2019. The blue, shaded region indicates the period where the US Food & Drug Administration (FDA) released the 2016 Federal Food, Drug and Cosmetic Act (FD&C Act).

To determine whether the frequency of each health symptom category mentioned on Reddit changes over time, we examined their longitudinal trend. Our results showed that the number of e-cigarette–related Reddit posts were steadily increasing over time since 2013 (Multimedia Appendix 2). We observed a spike in the temporal trend in June 2016 due to an increase in community discussion around the release of the Federal Food, Drug and Cosmetic (FD&C) Act on tobacco products and its substitutes, which prohibited e-cigarette and peripheral products to be sold to people aged under 18 years [7]. By examining the frequency of each health symptom category mentioned on Reddit over time, we observed that each health symptom category has a similar uptrend (Figure 1), which is similar to all e-cigarette–related posts. We observed that the Respiratory category mentions were consistently the highest among all health categories, whereas the Cancer category was the lowest. Spikes were also observed in the unnormalized temporal trend in June 2016 for all health categories due to the increased discussion on the FD&C Act. After normalization to the number of e-cigarette–related posts in each month, the percentage of health-related keywords mentions was relatively consistent over time, with one exception that the Psychological category had a noticeable growth from June 2016 to April 2019 (Figure 1).

### Sentiment Analysis of Posts With Mentions of Health Categories

Sentiment analysis was conducted applied to the health-related subset posts to understand users’ opinions regarding health symptoms in the discussion of e-cigarettes. Most health categories have a positive average sentiment score, whereas the Cancer category yields a negative average sentiment score (Table 1). For most health categories, the proportions of positive sentiment posts were significantly higher than the proportions of negative sentiment posts (*P*<.001), except for the Cancer category (Multimedia Appendix 3). Posts in the Cancer category had mostly negative sentiments, with over 70% of the posts identified with negative sentiment scores (*P*<.001). Example posters used in sentiment analysis are shown in Multimedia Appendix 1. For all categories, significantly more positive and negative sentiment posts were identified in comparison with neutral posts, suggesting that posts on Reddit usually had polarized opinions.

**Table 1:** Total post count and average sentiment score of posts in health categories.

Health category	Total post count	Average sentiment score
Respiratory	93,259	0.197
Cardiovascular	20,111	0.1889
Neurological	49,351	0.2862
Psychological	41,454	0.2104
Digestive	9123	0.1296
Mouth	62,011	0.2186
Throat	36,250	0.3062
Cancer	4983	−0.333

### Potential Associations Between E-liquid Flavors and Health Categories

The LDA topic model was applied to the health-related subset posts to reveal content-wise insights from the discussions on e-cigarette–related health symptoms. As we used the health subset, the majority of the contents were health related. Noticeably, 17 out of 100 topics were mostly related to e-liquid flavors (Textbox 1). A number of representative posts are also provided for positive and negative sentiment posts in each health category to show the topics of discussions on Reddit using a qualitative approach (Multimedia Appendix 1).

**Examples of top 100 topics observed from health-related posts on Reddit.**

**Health related (n=7)**

- Pain, symptom, relief, work, migraine, chronic, doctor, nausea, pill, and stomach
- Throat, vape, cold, allergy, irritate, sore, sensitive, sick, reaction, and irritation
- Smoking, vape, healthy, lung, alternative, bad, vaping, smoke, compare, and unhealthy
- Anxiety, depression, medication, attention-deficit/hyperactivity disorder, med, calm, panic attack, mental, symptom, and anxious
- Lung, cancer, die, kill, vape, death, tar, smoking, year, and chronic obstructive pulmonary disease
- Feel, vape, headache, sick, give, head, buzz, make, stomach, and feeling
- Cough, lung, asthma, breath, breathe, breathing, inhaler, clear, phlegm, and smoke

**Flavor related (n=10)**

- Juice, flavor, menthol, vendor, taste, order, vapor, bottle, sample, and Mt Baker Vapor, a major flavor concentrate wholesale for e-liquid DIY (MBV)
- Flavor, taste, strawberry, sweet, candy, favorite, fruit, juice, milk, and custard
- Vape, “flavour,” “vapour,” liquid, find, bit, UK, good, give, and taste
- Hit, throat, harsh, smooth, propylene glycol (PG), ratio, high, burn, vegetable glycerin, strong
- Flavor, taste, tongue, vaper, juice, strong, sweet, tasting, sense, and burn
- High, tetrahydrocannabinol, strain, cannabidiol, effect, anxiety, edible, cannabis, indica, and sativa
- Lung, popcorn, diacetyl, chemical, case, cigarette, inhale, amount, juice, and find
- Liquid, ingredient, propylene glycol, flavoring, safe, chemical, nicotine, e-liquid, PG, and flavor
- Flavor, taste, strawberry, sweet, candy, favorite, fruit, juice, milk, and custard
- Flavor, sweet, recipe, mix, taste, note, The Flavor Apprentice, a major flavor concentrate wholesale for e-liquid DIY (TFA), cream, cap, and candy

**Figure 2:**
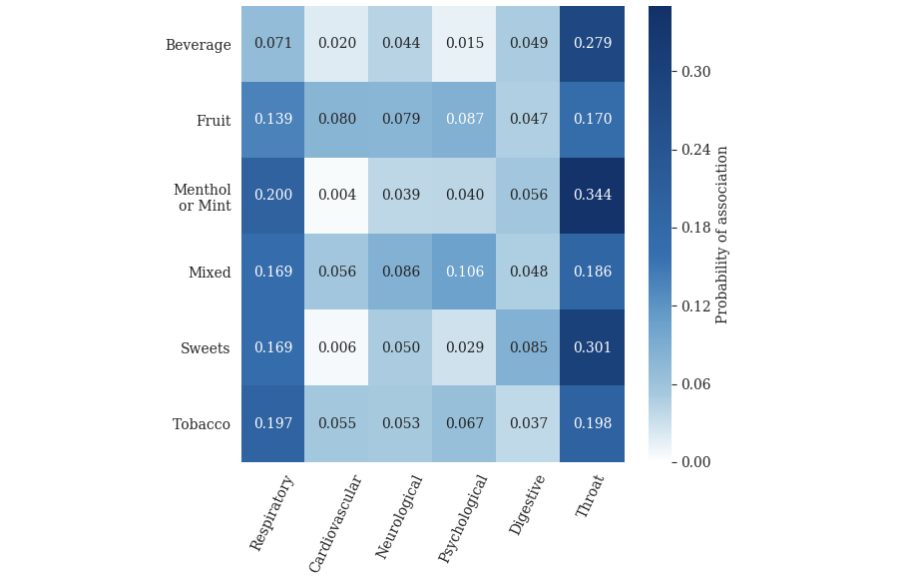
Heatmap of the estimated probabilities of associations between e-liquid flavors and health symptoms on Reddit.

The probability of each health category being simultaneously mentioned with e-liquid flavors was calculated from GEE models to examine the potential associations of e-liquid flavors with the reporting of health symptoms. The associations between some e-liquid flavors and some health symptoms were not investigated due to small sample sizes, which left us with the associations between six e-liquid flavors and six health symptoms (Figure 2). As shown in Figure 2, the Respiratory category had high probabilities of association with most flavors, including Menthol or Mint (0.200), Tobacco (0.197), Sweet (0.169), and Mixed (0.169). The Fruit flavor category (0.080) had a relatively higher probability of association with the Cardiovascular category than the other flavor categories. Both the Fruit (0.079) and Mixed (0.086) flavor categories had a relatively higher probability of association with the Neurological category than other flavor categories. The Mixed flavor (0.106) category had a relatively higher probability of association with the Psychological category than other flavors. The Sweet flavor (0.085) category had a relatively higher probability of association with the Digestive category than other flavors. The Throat category had relatively high probabilities of association with all flavor categories, with Menthol or Mint (0.344), Sweet (0.301), and Beverage (0.279) being the highest.

To test whether any e-liquid flavors have significantly higher probabilities of association with a certain health category than other flavors, we performed pairwise comparisons among e-liquid flavor categories on their associations within each health category (Multimedia Appendix 4). The Tobacco flavor category had a significantly higher probability of association with Respiratory than both Fruit and Beverage flavors. Both the Menthol or Mint and Mixed flavor categories had significantly higher probabilities of association with the Respiratory category than Beverage. Fruit flavor had higher probabilities of association with the Cardiovascular category than Menthol or Mint and Sweet. In the Neurological category, Mixed flavor had a significantly higher probability of association than the Tobacco flavor. For the Psychological category, Mixed flavor had a significantly higher probability of association than other flavor categories, except for Fruit flavor. Sweet flavor had higher probabilities of association than tobacco in the Digestive category. In the Throat category, a number of flavor categories had significantly higher probabilities of association than other categories (Menthol or Mint over Fruit, Mixed, and Tobacco; Sweet over Mixed and Tobacco; and Beverage over Fruit). No significant pairwise comparisons were observed between e-liquid flavors for the Cancer or Mouth category.

## Discussion

### Principal Findings

By examining the frequency of health keywords mentioned on Reddit, we showed that Respiratory was the most dominant health category, followed by Mouth, Throat, and Neurological categories. A previous social media study showed that the Mouth and Throat (combined), Respiratory, and Neurological health categories were the most frequently mentioned health symptoms on Reddit [25]. These frequency differences could be a result of different health-related keywords used, as our study included more keywords (n=23) in the Respiratory category than the mentioned previous study (n=4). E-cigarette consumption could lead to decreased exhaled nitric oxide, increased respiratory impedance, and increased flow respiratory resistance after short-term e-cigarette use, all of which are indications of immediate adverse physiological effects [26]. Adolescent e-cigarette users have increased rates of chronic bronchitis symptoms [9]. Using the PATH Wave 2 national survey data, e-cigarette use has been shown to be associated with wheezing and other related respiratory symptoms [10]. A recent cross-sectional study using the combined Behavioral Risk Factor Surveillance System 2016 and 2017 national survey data showed a significant association between e-cigarette use and asthma in US adults who never smoked combustible cigarettes [11]. The dominant temporal trend of the Respiratory category shown in our results, along with the previously reported effects of e-cigarettes on respiratory symptoms, suggests that respiratory diseases are highly related to e-cigarette use.

Our study analyzed users’ opinions on e-cigarettes from posts containing mentions of health keywords using sentiment analysis. Most health categories were dominated by positive sentiment posts, except that the Cancer category was dominated by negative sentiment posts. These results indicated a generally positive opinion toward e-cigarettes when Reddit users mentioned health-related keywords during discussion. As a forum consists of mostly teenagers and young adults, most users seem to advocate novel products such as e-cigarettes. This demographic characteristic could contribute to the average positive sentiments. In terms of the average negative sentiment on the Cancer category, we found that most posts discussed about the anecdotes of relatives or friends contracting cancer in the past with consumption of conventional tobacco products. This is reasonable considering the relatively young age of majority of the Reddit users, as cancer is not a prevalent disease in this age group (age: 13 to 32 years) [27]. We also noticed that a great proportion of posters were comparing e-cigarettes with conventional tobacco products, most of whom argued about the benefits of the former over the latter, thus contributing to the rather positive sentiment of most health keyword categories. A previous investigation on health-related effects reported by e-cigarette users in online forums also identified positive effects on the respiratory system, which is consistent with our study [21]. The sentiment analyses from this study are consistent with the sentiment analyses from a previous study using Reddit posts, which found a 60.7% provaping rating using textual analysis [17]. A recent investigation on online forum posts related to the health effects associated with e-cigarettes from 2008 to 2015, however, found that negative sentiment posts were dominant in almost all health categories [28]. This discrepancy might be due to the differences in the time frame, sentiment analysis methods, and data sources. Overall, the average positive sentiment scores across most categories reflect the provaping attitude of teenagers and young adults.

This study investigated the potential associations between e-liquid flavors and the reporting of health symptoms. Other than the Mouth and Cancer categories, some e-liquid flavor categories were identified to have significantly higher association with some health symptom categories compared with other flavor categories. For example, the Mixed flavors had a higher association with the Psychological category than other flavors except Fruit, suggesting that the Mixed e-liquid flavors might lead to a higher risk of psychological symptoms than other flavors. Using monocytes, one previous study showed that mixed flavors had greater cytotoxicity and higher levels of reactive oxygen species than individual flavors, which suggests that mixed flavors might be more harmful to e-cigarette users in psychological symptoms [29]. For the Throat category, Menthol or Mint had higher associations than other flavors, which is consistent with the previous finding that Menthol or Mint flavors lead to strong throat hit. For the Respiratory category, Menthol or Mint and Tobacco flavors had higher associations than other flavors, but this needs further exploration through both epidemiological and experimental studies. Previous studies have shown that some flavoring chemicals such as acetoin (in buttery flavor) and maltol (in candy flavor) can induce inflammatory responses and impair epithelial barrier function in human bronchial epithelial cells [30]. It has been shown that diacetyl, contained in buttery or creamy e-liquid [31,32], might be associated with respiratory disorders [33,34]. Cinnamaldehyde in cinnamon-flavored e-liquid has been shown to correlate with respiratory diseases and lung irritations [35-37]. In addition, benzaldehyde in cherry flavor and furfural in sweet flavor might lead to the irritation of respiratory airways [38]. Our results also showed a high probability of association (0.14-0.17) between Fruit, Mixed, and Sweet flavors and respiratory symptoms, which is consistent with previous studies. Although these associations between e-liquid flavors and health categories require further clinical validation, our results provided a systematic investigation on the association of e-liquid flavors and health symptoms using social media data and some valuable guidance for further studies.

### Limitations

Due to the characteristics of social media platforms, the user demographic information (including age, gender, etc.) as well as other factors associated with e-cigarettes such as Propylene Glycol/Vegetable Glycerin ratio and nicotine concentrations were not included in our data analysis. In addition, the Reddit data provide information from users’ perspectives, not from controlled laboratory experiments or clinical trials. Although such data provide us with the first-hand experience of e-cigarette users, they need to be further validated by well-designed laboratory or clinical studies. The measures including e-liquid flavors and health symptoms in this study were just mentions by Reddit users, which did not necessarily mean that the users were using these e-liquid flavors or having these health symptoms. In addition, as e-cigarettes have become increasingly popular in recent years, the research field in e-cigarettes has rapidly evolved. More health effects related to e-cigarettes have been identified. The new research findings might affect people’s opinions on e-cigarettes and their association with health symptoms. Meanwhile, a scare story related to e-cigarettes might also cause a flurry of health posts. This analysis did not consider the effects of this evolvement, which we will explore in our future studies.

### Conclusions

Using social media data from Reddit, our study quantitatively measured potentially different associations between e-liquid flavors and health symptoms. Temporal analysis revealed that mentions of keywords in most health categories were increasing, as the Psychological category had the most significant increase. Sentiment analysis showed that most health categories have a positive average sentiment score, whereas the Cancer category yields a negative average sentiment score. This study also investigated the potential associations between the reporting of health symptoms and e-liquid flavors. Other than the Mouth and Cancer categories, some e-liquid flavor categories were identified to have a significantly higher association with some health symptom categories compared with other flavor categories. With e-cigarette being prevalent worldwide and more e-liquid flavors being available, the health risks associated with e-cigarettes with different flavors should be further investigated. The results from this study could provide guidelines for future clinical and social media studies on the potential associations between e-liquid flavors and health symptoms as well as valuable guidance in future research on flavored e-cigarettes.

## Appendix

Multimedia Appendix 1 Supplemental Tables.

Multimedia Appendix 2 Monthly e-cigarette-related Reddit posts count from January 2013 to April 2019.

Multimedia Appendix 3 Proportion of positive, neutral and negative sentiment posts in each health categories.

Multimedia Appendix 4 Probabilities of the association between e-cigarette flavors and health categories. Error bars represent the upper halve of 95% CI.

## Abbreviations

e-cigarettes: electronic cigarettes
e-liquid: electronic cigarette liquid
ENDS: electronic nicotine delivery system
FD&C: Federal Food, Drug and Cosmetic
FIM: Frequent Itemset Mining
GEE: generalized estimating equation
LDA: Latent Dirichlet Analysis
PATH: Population Assessment of Tobacco and Health
VADER: Valence Aware Dictionary and sEntiment Reasoner
