# Supplementary material for: A Social Media Study on the Associations of Flavored Electronic Cigarettes With Health Symptoms: Observational Study
Source: J Med Internet Res. 2020 Jun 22;22(6):e17496. doi: 10.2196/17496 (PMC7338924; doi:10.2196/17496)
Supplement: Multimedia Appendix 1 [file jmir_v22i6e17496_app1.docx]

**Supplemental Tables**

**Supplemental Table 1: Accuracy of filtering with top ten flavor-related keywords**

| **Flavor Keyword** | **Accuracy of Filtering** |
| --- | --- |
| Fruit | 0.84 |
| Tobacco | 0.91 |
| Coffee | 0.76 |
| Menthol | 0.87 |
| Mint | 0.83 |
| Apple | 0.72 |
| Strawberry | 0.85 |
| Vanilla | 0.87 |
| Melon | 0.82 |
| Banana | 0.86 |

**Supplemental Table 2: Health Symptom Keyword Categorization**

| **Health Category** | **Keywords** |
| --- | --- |
| **Cardiovascular**  **(n=16)** | heart, cholesterol, CHF, stroke, angina, chest pain, chest tightness, high blood pressure, low blood pressure, congestive heart failure, heart attack, myocardial infraction, coronary heart disease, cardiovascular disease, blood clots, heart rate |
| **Respiratory**  **(n=23)** | wheezing, cough, coughing, coughed, coughs, COPD, emphysema, asthma, asthmatic, respiratory, lung, lungs, shortness of breath, dry cough, chronic bronchitis, wet lung, popcorn lung, acute bronchitis, pneumonia, cystic fibrosis, interstitial lung disease, mesothelioma, sleep apnea |
| **Neurological**  **(n=15)** | seizure, seizures, headache, dizzy, dizziness, fatigue, fatigued, numb, numbness, tingle, tingling, tingles, sensitive, sensitivity, sleeping disorder |
| **Cancer**  **(n=13)** | lung cancer, mouth cancer, liver cancer, colon cancer, rectum cancer, throat cancer, larynx cancer, stomach cancer, pancreatic cancer, bladder cancer, kidney cancer, cervix cancer, leukemia |
| **Psychological**  **(n=8)** | depression, depressed, unrested, irritable, stress, dysthymia, anxiety, ADHD |
| **Mouth**  **(n=22)** | dentist, teeth, tongue, periodontal, toothache, mouth pain, gum bleeding, looser teeth, oral lesions, oral health, tooth decay, gum disease, gingivitis, root planning, dry mouth, loss of taste, vaper’s tongue, lips, gum, gums, dry socket, cavities |
| **Throat**  **(n=3)** | esophagus, throat, throat hit |
| **Digestive**  **(n=9)** | digestive, stomach, stomachache, heartburn, eating disorder, vomit, vomiting, vomited, diarrhea |
| **Other**  **(n=36)** | nausea, nauseous, nauseated, lightheaded, light-headed, kidney, kidneys, bladder, colon, liver, skin, diabetes, ulcer, ulcers, arthritis, aching, osteoporosis, dehydrate, dehydrates, dehydrated, dehydration, hurt, hurting, hurts, irritate, irritates, irritated, irritation, inflammation, stamina, vision loss, macular degeneration, sinus, sinuses, phlegm, acne |

**Supplemental Table 3: Flavor Keyword Categorization**

| Flavor Category | Flavor Subcategory | List of Specific Flavors |
| --- | --- | --- |
| Fruit | Berry | Wildberry, Currant, Blackcurrant, Blackberry, Grape, Raspberry, Blueberry, Strawberry, etc. |
|  | Tropical | Mango, Lychee, Guava , Passion Fruit, Pineapple, etc. |
|  | Citrus | Grapefruit, Lime, Orange, Lemon, etc. |
|  | Melon | Cantaloupe, Honeydew, Melon  Watermelon, etc. |
|  | Mixed Fruits | Mango Apricot, Apple Melon, Nana Berry, etc. |
|  | Others | Pomelo, Papaya, Apricot, Dragon Fruit, Pomegranate, Cucumber, Kiwi, Pear, Cherry, Peach, Coconut, Banana, Apple, etc. |
| Sweets | Dessert | Mochi, Pie, Waffle, Donut, Mixed, Cake, S'more, Muffin, Ice Cream, Cream, Custard, Macaron, Granola, Pastry, Meringue, Bread, Cheesecake, Cookie, etc. |
|  | Candy | Lollypop, Mixed, Jelly Bean, Gummy Bear, Cotton Candy, Marshmallow, Bubble Gum, Chocolate, etc. |
|  | Others | Cereal, Honey, Caramel, etc. |
| Beverage | Coffee | Latte, Mocha, Cappuccino, Espresso, Coffee |
|  | Tea | Chai, Tea, etc. |
|  | Juice | Limeade, Lemonade, Apple Juice, etc. |
|  | Milk | Yogurt, Milkshake, Milk |
|  | Soft Drinks | Cola, Coke, Soda, etc. |
|  | Others | Energy Drink, Smoothie, etc. |
| Tobacco | Tobacco | Classic Tobacco, Virginia Tobacco, Cigar, etc. |
| Menthol/Mint | Menthol | Menthol |
|  | Mint | Mint, Peppermint, Spearmint |
| Mixed | Mixed | Fruit + Mint, Fruit+Sweets, Fruit+Beverage, Fruit+Beverage, Sweets+Other, Sweets+Mint, Sweets+Tobacco, Fruit+Tobacco, etc. |
| Others | Alcohol | Margarita, Whiskey, Rum, Bourbon, Cocktail, etc. |
|  | Nuts | Walnut, Pecan, Pistachio, Hazelnut, Almond, Peanut Butter, etc. |
|  | Spice | Vanilla, Cinnamon, etc. |
|  | Others | Pure VG, Pure PG, PG/VG, etc. |

**Supplemental Table 4: Example posts of health categories, with positive and negative sentiments**

| **Health Category** | **Sentiment** | **Example posts** |
| --- | --- | --- |
| **Cardiovascular** | Positive | Can I recommend the Magic Flight Launch Box, to hopefully relieve any future *heart attacks*? Handheld, you can re-smoke or eat the bud when done vaping, and it's pretty badass… |
|  | Negative | … I was vaping some 24mg, so it was a little strong but i think I may have gotten some juice on me and it soaked in. Nicotine poisining causes hyper tension so my *high blood pressure* prolly didn't help the situation. |
| **Respiratory** | Positive | Not nearly as bad as smoking tobacco but a vape might be a wise choice if you begin to notice a *shortness of breath*. |
|  | Negative | It took me 2ish days for my lungs to adjust to dealing with vaping; I *coughed* on every hit up 'til that point… |
| **Neurological** | Positive | I went for my first bike ride of the year yesterday and I could feel a massive difference in my heart and lungs. No *fatigue*, no gasping, no chest burn… but at the end of the day e-cigs are better in every conceivable way. |
|  | Negative | Could be palate *fatigue* aka vaper's tongue…Some people vape unflavored juice for a while until their tastebuds recover. |
| **Cancer** | Positive | …The vapor is so cool, it's like I'm not even vaping at all. Kind of annoying. Consistent as hell, though. I haven't burned it once, unlike the Vivi Nova, which I am sure is giving me *lung cancer* from all the wick I've smoked. |
|  | Negative | Health-wise, although you will be reducing the risk of *lung cancer*, you're still inhaling burnt plant matter. |
| **Psychological** | Positive | …One of the best decisions I've made, as vaping has really evened me out (*anxiety* and the likes.) |
|  | Negative | Have you been vaping long? 1-2ml of 11mg in a day is not a whole lot…some get *stressed* by the fact that they arent really sure how much nicotine they are getting and become afraid that they are gonna overdose. |
| **Mouth** | Positive | …This makes a vacuum in your mouth just strong enough to draw the vapor into your lungs. Impressively deep hits this way. |
|  | Negative | The thing is, I feel vaping isn't fantastic for *teeth* so shoveling sugar in my mouth regularly throughout the day is a pretty bad idea. |
| **Throat** | Positive | Vaping is by far the best and most effective methods of quitting I have tried… My lungs are clean and I no longer have to clear my *throat* constantly. |
|  | Negative | PG always left a prickly feeling in my *throat*, almost spicy. I do have problems with vaping causing me to hiccup though. |
| **Digestive** | Positive | I would wake up ,even hungover *vomiting*, and still go have a cig. The key is to sit down have that cup of joe in the AM and Vape that Nicotine fix… |
|  | Negative | If you chain-vape too-high nicotine content, you're first going to get a headache and get dizzy. Then you're start *vomiting*, get *diarrhea*, and break into a cold sweat. |
