# Supplementary figures and images for: A Social Media Study on the Associations of Flavored Electronic Cigarettes With Health Symptoms: Observational Study
Source: J Med Internet Res. 2020 Jun 22;22(6):e17496. doi: 10.2196/17496 (PMC7338924; doi:10.2196/17496)

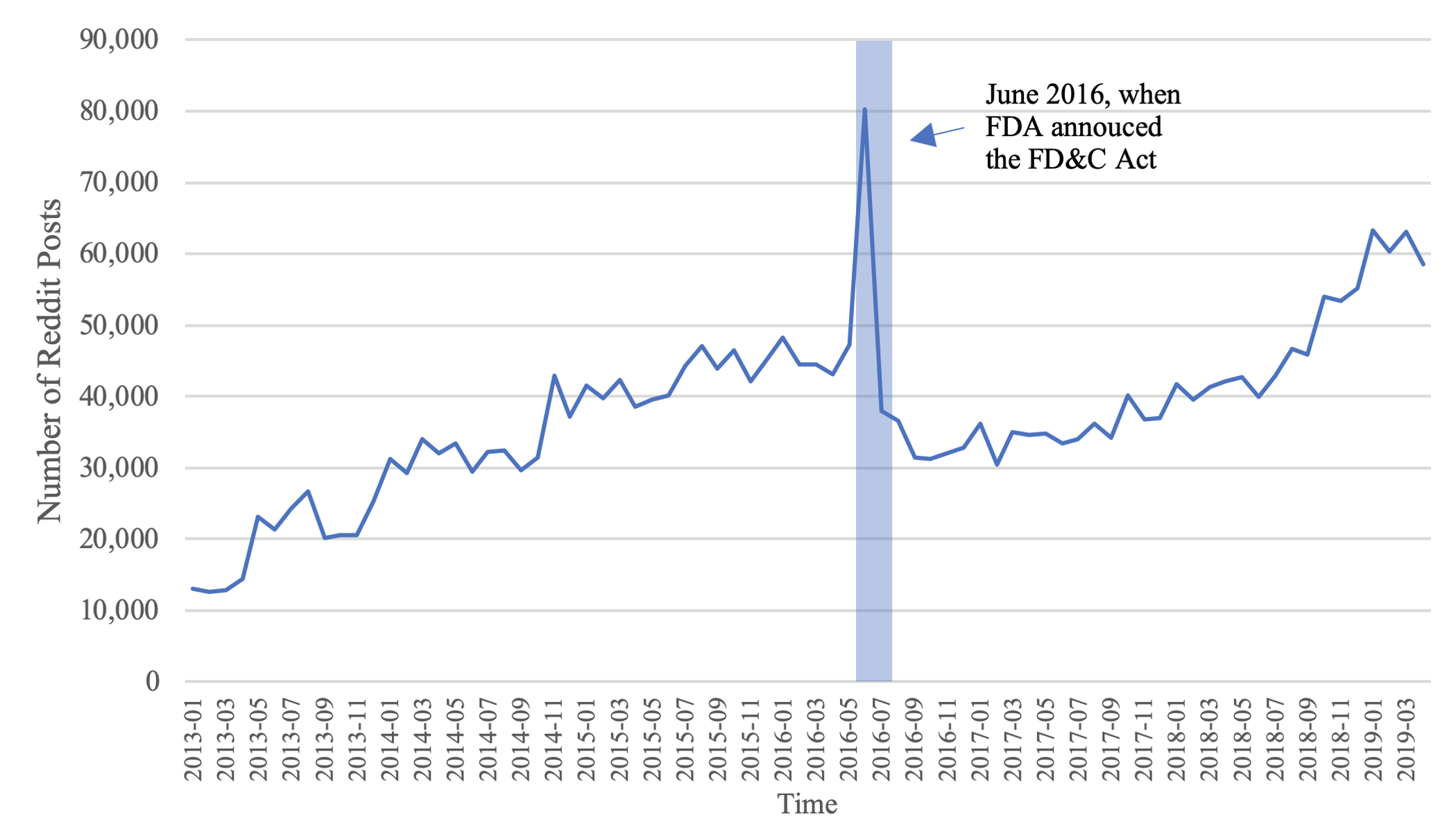

Supplement: Multimedia Appendix 2 [file jmir_v22i6e17496_app2.png]

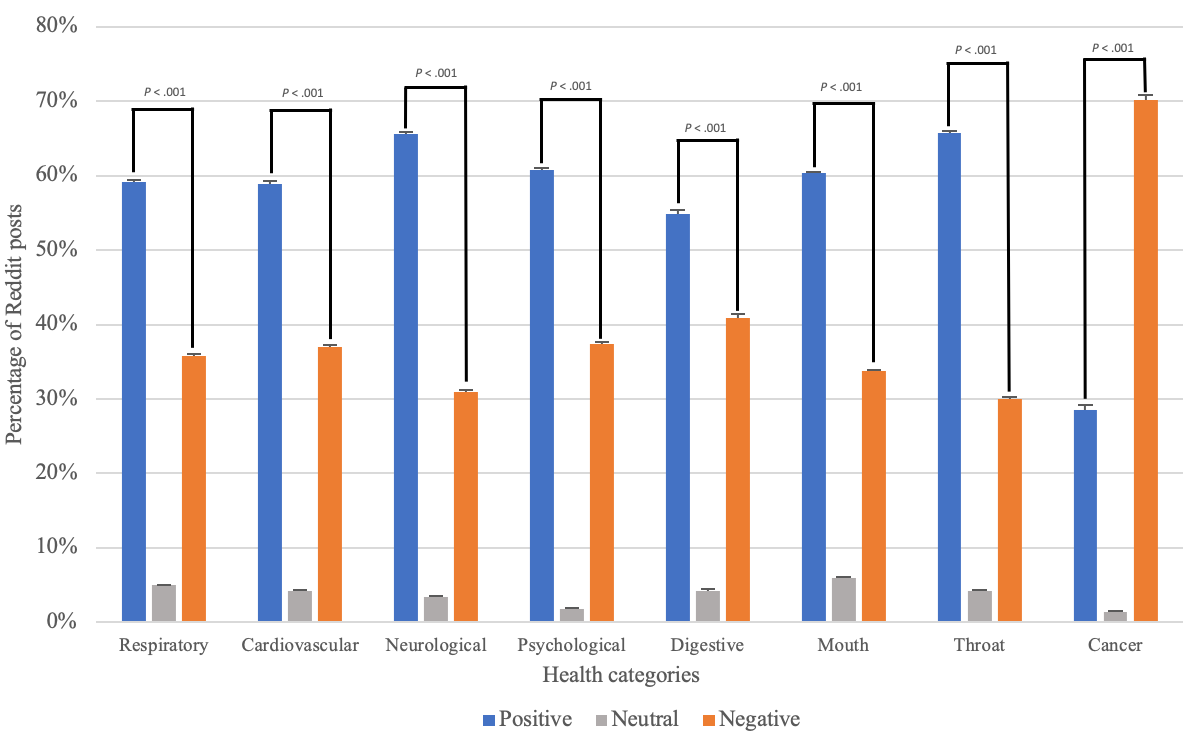

Supplement: Multimedia Appendix 3 [file jmir_v22i6e17496_app3.png]

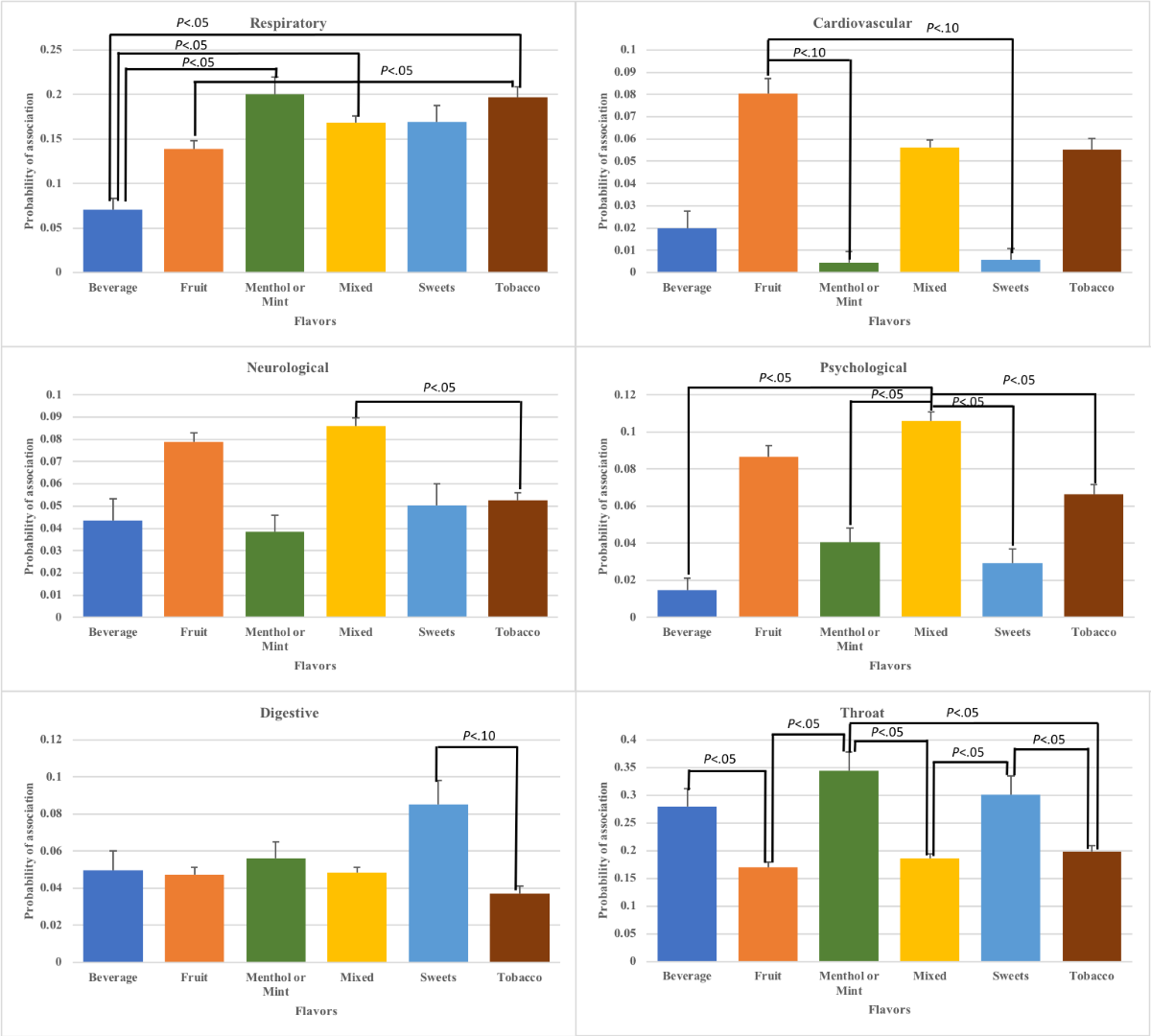

Supplement: Multimedia Appendix 4 [file jmir_v22i6e17496_app4.png]
